# Maternal peripheral blood natural killer cells incorporate placenta-associated microRNAs during pregnancy

**DOI:** 10.3892/ijmm.2015.2157

**Published:** 2015-03-27

**Authors:** YOICHI ISHIDA, DONGWEI ZHAO, AKIHIDE OHKUCHI, TOMOYUKI KUWATA, HIROSHI YOSHITAKE, KAZUYA YUGE, TAKAMI TAKIZAWA, SHIGEKI MATSUBARA, MITSUAKI SUZUKI, SHIGERU SAITO, TOSHIHIRO TAKIZAWA

**Affiliations:** 1 Department of Obstetrics and Gynecology, Jichi Medical University, Tochigi 329-0498, Japan; 2 Department of Molecular Medicine and Anatomy, Graduate School of Medicine, Nippon Medical School, Tokyo 113-8602, Japan; 3 Department of Obstetrics and Gynecology, Faculty of Medicine, University of Toyama, Toyama 930-0194, Japan

**Keywords:** microRNA, chromosome 19 microRNA cluster, mRNA, maternal peripheral blood, natural killer cell, human placenta

## Abstract

Although recent studies have demonstrated that microRNAs (miRNAs or miRs) regulate fundamental natural killer (NK) cellular processes, including cytotoxicity and cytokine production, little is known about the miRNA-gene regulatory relationships in maternal peripheral blood NK (pNK) cells during pregnancy. In the present study, to determine the roles of miRNAs within gene regulatory networks of maternal pNK cells, we performed comprehensive miRNA and gene expression profiling of maternal pNK cells using a combination of reverse transcription quantitative PCR (RT-qPCR)-based miRNA array and DNA microarray analyses and analyzed the differential expression levels between first- and third-trimester pNK cells. Furthermore, we constructed regulatory networks for miRNA-mediated gene expression in pNK cells during pregnancy by Ingenuity Pathway Analysis (IPA). PCR-based array analysis revealed that the placenta-derived miRNAs [chromosome 19 miRNA cluster (C19MC) miRNAs] were detected in pNK cells during pregnancy. Twenty-five miRNAs, including six C19MC miRNAs, were significantly upregulated in the third- compared to first-trimester pNK cells. The rapid clearance of C19MC miRNAs also occurred in the pNK cells following delivery. Nine miRNAs, including eight C19MC miRNAs, were significantly downregulated in the post-delivery pNK cells compared to those of the third-trimester. DNA microarray analysis identified 69 NK cell function-related genes that were differentially expressed between the first- and third-trimester pNK cells. On pathway and network analysis, the observed gene expression changes of pNK cells likely contribute to the increase in the cytotoxicity, as well as the cell cycle progression of third- compared to first-trimester pNK cells. Thirteen of the 69 NK cell function-related genes were significantly down-regulated between the first- and third-trimester pNK cells. Nine of the 13 downregulated NK-function-associated genes were *in silico* target candidates of 12 upregulated miRNAs, including C19MC miRNA *miR-512-3p*. The results of this study suggest that the transfer of placental C19MC miRNAs into maternal pNK cells occurs during pregnancy. The present study provides new insight into maternal NK cell functions.

## Introduction

Natural killer (NK) cells are large granular lymphocytes that function as effectors of innate immune activity and modulators of adaptive immune responses (1,2). NK cells kill stressed and abnormal cells, such as virus-infected cells and tumor cells, and can also produce cytokines. NK cell-mediated cytotoxicity and cytokine production are regulated by NK cell surface receptors, such as activating, inhibitory, adhesion, cytokine and chemotactic receptors, and the relative balance of these receptor-mediated signals fine-tunes the various biological activities of NK cells. NK cells circulate in the peripheral blood NK (termed pNK cells) (3). NK cells are also present in the uterus (4). In the human placenta, there are two distinct fetal maternal interfaces, the first is the decidua and the second is the intervillous space where maternal blood bathes the floating chorionic villous tree. A specific population of NK cells accumulates within the decidua. These NK cells, referred to as decidual NK (dNK) cells, are considered to play a pivotal role in placentation, including trophoblast invasion and uterine spiral artery remodeling (5,6). As pNK cells can come into direct contact with chorionic villi at the intervillous space, mechanisms of resistance to pNK cytotoxicity may be involved at the second interface. This second interface constitutes a privileged site where the secretion of placental microvesicles, including exosomes, into the maternal peripheral blood occurs. Hedlund *et al* reported that the human placenta secretes KLRK1 ligands via exosomes that induce the downregulation of the KLRK1 receptor on pNK cells, leading to a reduction in their cytotoxicity *in vitro* (7). The syncytiotrophoblast covering chorionic villi may evade NK cytotoxicity from these cells.

MicroRNAs (miRNAs or miRs) are small non-coding RNAs that play a pivotal role in post-transcriptional gene regulation by targeting the 3′-untranslated region (3′-UTR) of specific target mRNAs for endonucleolytic cleavage or translational repression (8). With regard to human NK cell miRNAs, genome-wide comparisons have been made for human lymphocytes subsets, including NK cells (9,10). Two studies have also reported the miRNA profiles of resting and cytokine-activated pNK cells using next-generation sequencing (11,12). Despite such progress, knowledge of the NK cell miRNA profiles and their physiological roles remain incomplete. Moreover, little is known about the miRNA-gene regulatory relationships that may be relevant for the functions of maternal NK cells during pregnancy.

In the present study, to determine the roles of miRNAs within gene regulatory networks of maternal pNK cells during pregnancy, we performed comprehensive miRNA and gene expression profiling of NK cells isolated from the peripheral blood of healthy pregnant females and analyzed these differential expression levels between first- and third-trimester pNK cells. We explored NK cell function-associated genes that were negatively correlated with miRNA expression levels and computationally predicted to be miRNA targets. Finally, we constructed a regulatory network for miRNA-mediated gene expression in pNK cells during pregnancy using miRNA and gene expression profiles.

## Materials and methods

### pNK cell isolation from pregnant females

Samples of peripheral blood were obtained from pregnant females after obtaining informed consent. For the comprehensive analysis of mRNA and gene expression profiles in pNK cells, samples were obtained from the same healthy pregnant females during the first (gestational age, 7–11 weeks), second (19–23 weeks) and third (36–38 weeks) trimesters of gestation (n=5 each), and from other females who had a normal pregnancy 4 days following delivery (n=5). For the validation of miRNA expression levels by reverse transcription quantitative PCR (RT-qPCR, real-time PCR) in pNK cells, a different set of experiments with other healthy pregnant females was performed; samples were obtained from the same females in the first, second and third trimesters of gestation (n=5 each), and from other females who had a normal pregnancy 4 days following delivery (n=5). The study protocols were approved by the Ethics Committees of Jichi Medical University (Tochigi, Japan) and Nippon Medical School (Tokyo, Japan). Peripheral blood mononuclear cells were isolated from heparinized venous blood using Lymphoprep (Axis-Shield PoC AS, Oslo, Norway) as previously described (13). NK cells were isolated from the peripheral blood mononuclear cells using the Dynabeads Untouched NK Cells kit (Invitrogen, Carlsbad, CA, USA) according to the manufacturer’s instructions.

Total RNA within the cells was extracted using RNAiso reagent (Takara Bio, Inc., Shiga, Japan) according to the manufacturer’s instructions. The integrity of the RNA was determined using an Agilent 2100 Bioanalyzer (Agilent Technologies, Santa Clara, CA, USA); samples with an RNA integrity number >7 were used.

### Quantitative PCR-based array analysis of miRNAs

We performed real-time PCR-based array analysis to quantitatively and comprehensively examine the expression levels of 756 miRNAs in the pNK cells obtained from pregnant females. Total RNA from each specimen (each 30 ng) was reverse transcribed using Megaplex RT Primers (Applied Biosystems, Foster City, CA, USA). The cDNA was then pre-amplified using Megaplex PreAmp Primers (Applied Biosystems). The pre-amplified products were subjected to real-time PCR using TaqMan Array Human MicroRNA Cards (A and B, version 2.0) on a 7900HT Fast Real-Time PCR System (Applied Biosystems) according to the manufacturer’s instructions. The miRNA sequences were annotated using the Sanger database (miRBase), release 14. Data obtained with this assay were analyzed using RQ Manager 1.2 (Applied Biosystems). The relative Ct method (ΔΔCt method) was applied for the quantification of each miRNA expression level, as previously described (14). The data were normalized relative to the expression of *RNU6-2*.

### Real-time PCR analysis for the validation of miRNA expression levels

Real-time PCR for miRNA expression was carried out using TaqMan MicroRNA Assays on a 7300 Real-Time PCR System (both from Applied Biosystems). Briefly, total RNA (each 10 ng) was reverse transcribed with a High Capacity cDNA Reverse Transcription kit (Applied Biosystems). The RT products were subsequently subjected to real-time PCR using TaqMan 2X Universal PCR Master Mix (Applied Biosystems). To normalize the expression levels of the miRNAs, *SNORD44* was used as an endogenous internal control. Primers for *miR-517a-3p* (assay ID 002402) and *miR-518b* (assay ID 001156) were from Applied Biosystems.

### DNA microarray analysis

We performed microarray analysis of the gene expression levels in the first- and third-trimester pNK cells. Of the total RNA obtained, 20 ng was used in a labeling reaction with a Low-Input Quick Amp Labeling kit, one-color (Agilent Technologies), and the quality and yield of the labeled cRNA were evaluated on an Agilent 2100 Bioanalyzer. Gene expression profiling was conducted using an Agilent Microarray (Human GE 4×44K, version 2). The resulting signals were normalized relative to the 75th percentile signal intensity and spots with low reproducibility were omitted (coefficient of variation <50%). The processed data were filtered for P<0.05 (paired t-test) using the GeneSpring GX software (version 11.5; Agilent Technologies). The mRNA array data are publicly available (Gene Expression Omnibus, accession no. GSE64884; http://www.ncbi.nlm.nih.gov/geo/).

### Pathway and network analysis

Molecular networks of the selected molecules and specific pathways were analyzed by the Core Analysis of fold change for the third- vs. first-trimester pNK cell group in Ingenuity Pathway Analysis (IPA; www.ingenuity.com). A total of 715 NK cell function-related genes have been previously reported (1,15). Of these 715 NK cell function-related genes, 69 were included in the 1,173 differentially expressed genes (P<0.05) in the above-mentioned microarray data and were used for IPA. In addition to IPA, the miRNA.org database (August 2010 release; http://www.microrna.org/microrna/getMirnaForm.do) was also used to predict the targets of miRNAs.

### Statistical analysis

We conducted all analyses using the SPSS statistical software package (Windows version 20; IBM-SPSS, Inc., Chicago, IL, USA). The significance of between-group differences was assessed using ANOVA followed by Tukey’s test, or the Kruskal-Wallis test, and a value of P<0.05 was considered to indicate a statistically significant difference.

## Results

### Differential expression of miRNAs in pNK cells during and after pregnancy

**Figure 1:**
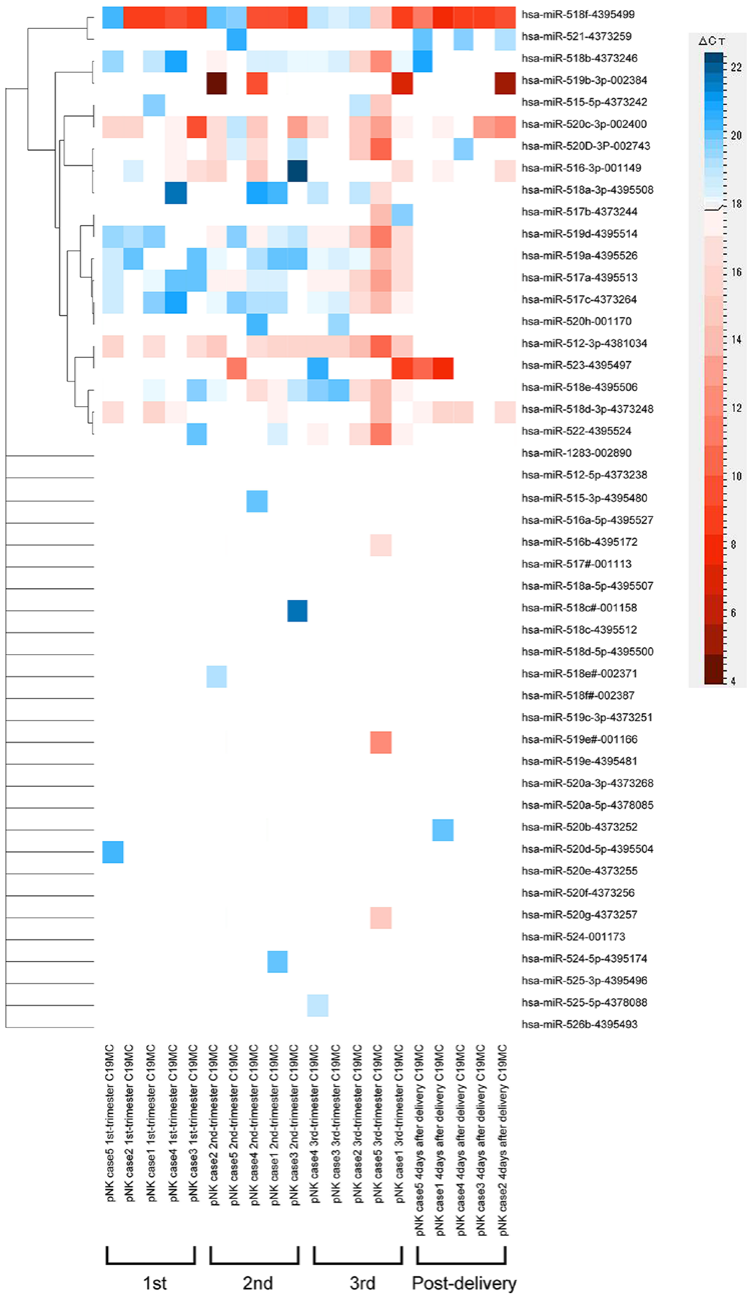
Clustering heat map of the chromosome 19 miRNA cluster (C19MC) miRNA expression profiles in peripheral blood NK (pNK) cells by real-time PCR-based array analysis. The samples at different time points of pregnancy [first (1st), second (2nd), and third (3rd) trimester of gestation, and post-delivery] are listed in columns, and the C19MC miRNAs in rows. The relative miRNA expression is depicted according to the color scale shown on the right. Red indicates higher abundance; blue indicates lower than median abundance; white indicates no detection.

We performed real-time PCR-based array analysis of the expression levels of 756 miRNAs in pNK cells at different time points of pregnancy (first, second and third trimester of gestation, and post-delivery). Among the 756 miRNAs examined, 293, 293, 294 and 325 miRNAs were detected in the pNK cells obtained from females in the first, second and third trimester of gestation, and post-delivery, respectively. Of the 47 mature chromosome 19 miRNA cluster (C19MC) miRNAs, which are expressed exclusively in the placenta (16–18), probed in the array cards, 12, 15, and 18 placental C19MC miRNAs (26, 32 and 38% of the C19MC miRNAs, respectively) were detected in the pNK cells obtained from the females in the first, second and third trimester of gestation, respectively (Fig. 1). By contrast, the number of detectable C19MC miRNAs decreased following delivery; only six C19MC miRNAs (13%) were detected in the pNK cells obtained from females following delivery (Fig. 1).

**Figure 2:**
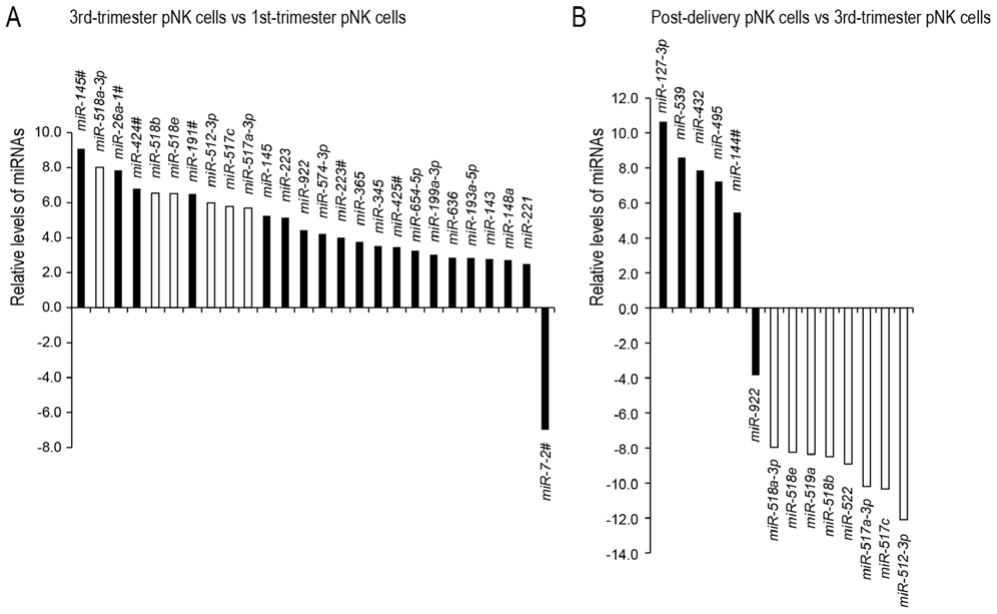
Differential expression of miRNAs in maternal peripheral blood NK (pNK) cells, as determined using a real-time PCR-based miRNA expression array. Differential profiles of miRNAs (Tukey’s test; P<0.05) in (A) third-trimester pNK cells in comparison with first-trimester pNK cells and (B) post-delivery pNK cells in comparison with third-trimester pNK cells. Data were normalized relative to *RNU6-2*. The x-axis indicates the miRNA rank from the most upregulated to the most downregulated. The y-axis represents the log fold change in the miRNA level relative to the corresponding control cells. White bars indicate chromosome 19 miRNA cluster (C19MC) miRNAs. An array data set of the differential expression of miRNAs is available upon request.

A comparison of the miRNA expression profiles revealed that 25 miRNAs were significantly upregulated in the third-compared to the first-trimester pNK cells (P<0.05; Fig. 2A). The upregulated miRNAs included six C19MC miRNAs (24% of the upregulated miRNAs). Only one miRNA, *miR*-7-2# [currently defined as *miR-7-2-3p* in miRBase release 21 (http://www.mirbase.org/)], was significantly downregulated in the third-trimester pNK cells compared to those in the first trimester. Subsequently, a comparison of the miRNA expression profiles between the third-trimester and post-delivery pNK cells identified nine miRNAs, including eight C19MC miRNAs (89% of the downregulated miRNAs), which were significantly downregulated in the post-delivery pNK cells compared to the third-trimester pNK cells (Fig. 2B). Five miRNAs were significantly upregulated in the post-delivery pNK cells compared to the third-trimester pNK cells.

**Figure 3:**
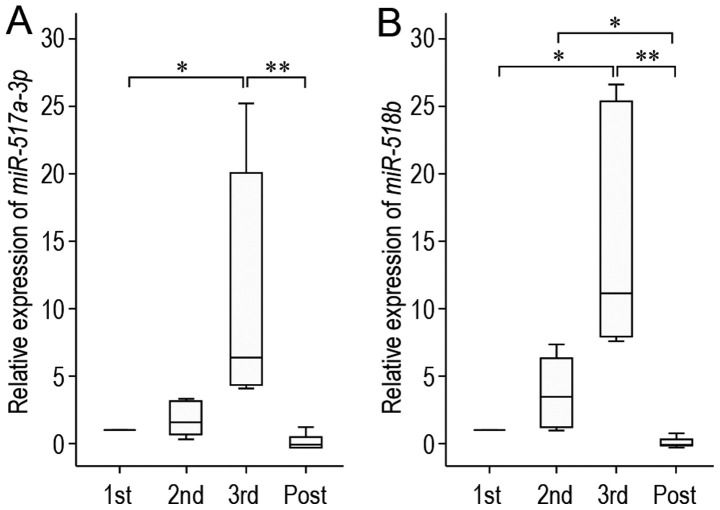
Validation of chromosome 19 miRNA cluster (C19MC) miRNAs in peripheral blood NK (pNK) cells by real-time PCR analysis. Transferred (A) *miR-517a-3p* and (B) *miR-518b* were measured by real-time PCR in pNK cells in the first (1st), second (2nd), and third (3rd) trimesters of gestation and post-delivery (Post) (n=5 each). Data were normalized relative to *SNORD44*. Expression in first-trimester pNK cells was defined as 1. Lines inside boxes denote medians, boxes represent the interquartile range, and whiskers extend to the most extreme values within 1.5× the interquartile range. Kruskal-Wallis test; *P<0.05, **P<0.001.

To confirm the differential C19MC miRNA expression identified by real-time PCR-based array assay, we investigated the expression of *miR-517a-3p* and *miR-518b* in the pNK cells at different time points of pregnancy by RT-qPCR (Fig. 3) as these miRNAs were included in the 25 upregulated miRNAs of the third-trimester pNK cells. A different set of experiments with other pregnant females was performed. In the pNK cells obtained during pregnancy, *miR-517a-3p* and *miR-518b* were detected; these C19MC miRNAs were significantly upregulated in a gestational stage-dependent manner (Fig. 3). On the other hand, the levels of *miR-517a-3p* and *miR-518b* were significantly decreased at 4 days following delivery (Fig. 3). These results suggest that the transfer of placental C19MC miRNAs into maternal pNK cells occurs during pregnancy.

### Comparison of gene expression between first- and third-trimester pNK cells

Using DNA microarray analysis, we assessed differentially expressed genes in the pNK cells during pregnancy to identify genes whose expression levels negatively correlated with those of the miRNAs. At the transcriptional level, the third-trimester pNK cells differed markedly from the first-trimes ter pNK cells. DNA microarray analysis revealed the differential expression of 1,173 genes among the 27,958 transcripts examined in this study (P<0.05) (a data set of the differential expression of 1,173 genes is available upon request). Among the 1,173 genes, 264 genes were significantly downregulated in the third- compared to the first-trimester cells. On the other hand, the expression of 909 genes was found to be significantly increased in the third- compared to the first-trimester cells.

Furthermore, we focused on the 715 genes which have been previously reported to be related to NK cell functions (i.e., cytolytic pathway, cytokines/chemokines and their receptors, secretory signature, cell surface/adhesion molecules, cell cycle and proliferation and signal transduction) (1,15). Among these 715 genes, 69 NK cell function-related genes were differentially expressed between the first- and third-trimester pNK cells (P<0.05); these included genes encoding NK cell surface and adhesion molecules, as well as cell cycle-related genes (Table I). Thirteen genes were significantly downregulated in the third- compared to the first-trimester cells. Of note, as regards cell surface/adhesion molecules, killer-cell immunoglobulin-like receptor (KIR) genes (e.g., *KIR2DL4*) showed significantly a decreased expression in the third- compared to the first-trimester cells. By contrast, the expression levels of leukocyte Ig-like receptor (LILR) genes (e.g., *LILRB2*) in the third-trimester cells were significantly higher than those in the first-trimester cells. With regard to cell cycle and proliferation, the third-trimester pNK cells showed a high-level expression of a group of genes involved in regulating cell cycle transitions (e.g., *CDK2* and *CCNA1*) (Table I).

### Bioinformatics analysis of functional pathways and network of the differentially expressed genes

**Figure 4:**
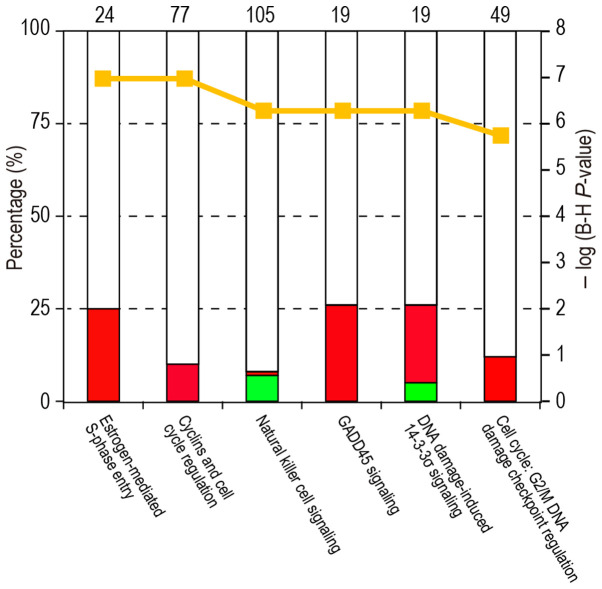
Ingenuity Pathway Analysis (IPA) canonical pathways most significantly changed in third-trimester peripheral blood NK (pNK) cells compared to first-trimester pNK cells. The stacked bar chart displays the percentage of genes that were upregulated (red), downregulated (green), and genes not overlapping with our data set (white) in each canonical pathway. The numerical value at the top of each bar represents the total number of genes in the canonical pathway. The secondary y-axis (right) shows the −log of P-value calculated by the Benjamini-Hochberg (B-H) method; the B-H method was used to adjust the right-tailed Fisher’s exact test P-value, which indicates the significance of each pathway.

We then used the IPA tool to search for molecular pathways associated with pNK cells during pregnancy. A data set of 69 NK cell function-associated genes mentioned above was included in the IPA Core Analysis that allowed us to interpret the data set in the context of biological processes, pathways and gene networks. The top canonical pathways and gene networks that were most significantly affected in the pNK cells during pregnancy are shown in Fig. 4. High-ranking canonical pathways included the NK cell signaling pathway (P=5.13×10^−7^), as well as cell cycle regulation pathways (e.g., estrogen-mediated S-phase entry pathway, P=1.02×10^−7^). The NK cell signaling pathway contained downregulated KIR genes (i.e., *KIR2DL4*, *KIR2DL5A*, *KIR2DS4* and *KIR3DL1*).

**Figure 5:**
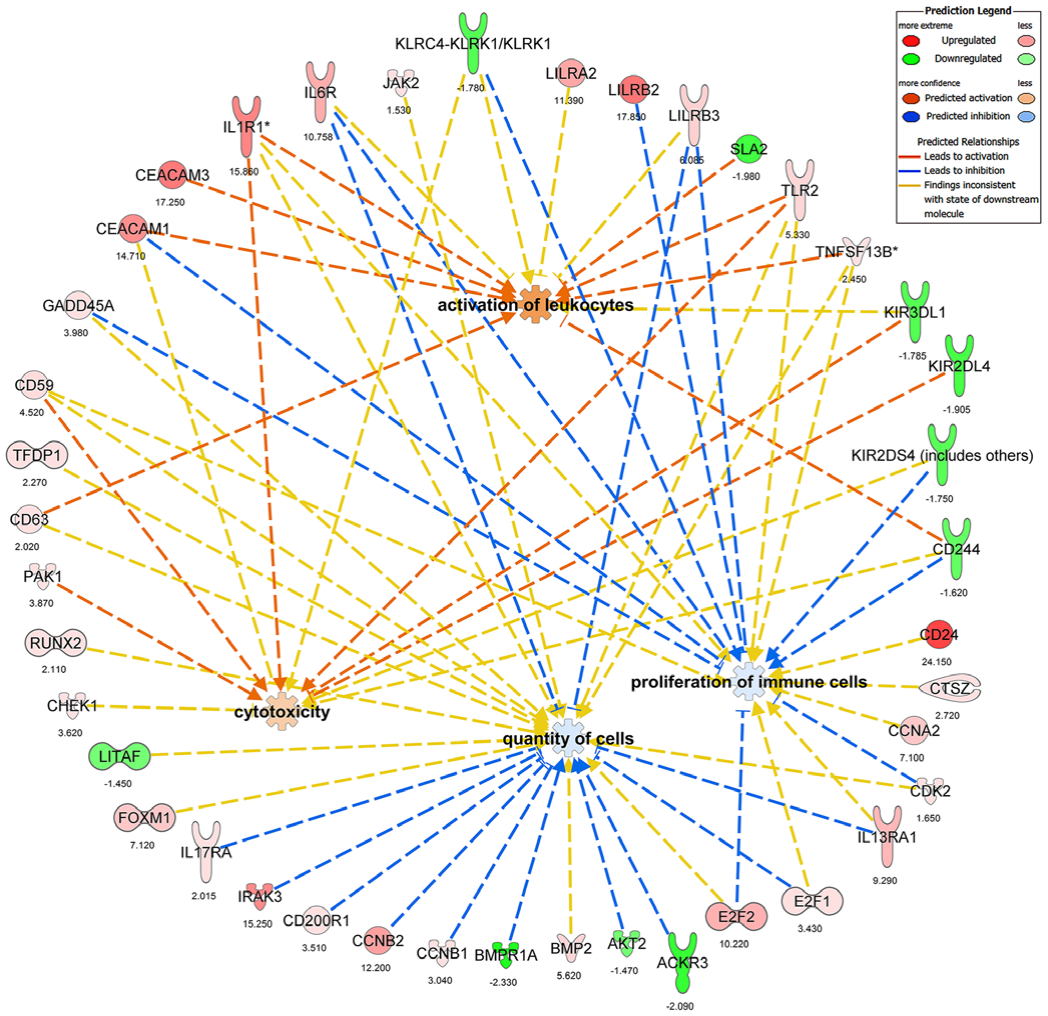
Ingenuity Pathway Analysis (IPA) Downstream Effects Analysis-based network associated with high-ranking natural killer (NK) cell functions. The NK cell functions (‘immune cells’, ‘quantity of cells’, ‘activation of leukocytes’, and ‘cytotoxicity’) in the IPA-defined Diseases and Bio Functions category were biologically relevant to a set of 41 NK cell function-related genes that were differentially expressed between first- and third-trimester peripheral blood NK (pNK) cells. Edges and nodes are color-coded based on the predicted relationship as indicated in the Prediction Legend. Numbers below symbols represent the fold changes in the transcript level of third- relative to first-trimester pNK cells.

We found that 136 gene-related functions with a minimum of ten genes per category in the IPA defined Diseases and Bio Functions category (an IPA data set of the 136 functions is available upon request). Moreover, we filtered the 136 functions by NK cell context with a minimum of ten genes per category. The filtering found 16 functions for NK cells; 54 genes were selected among the 69 NK cell function-associated genes (Table II). Among the 16 functions, the top scoring functions were the proliferation of immune cells (rank 8 relative to the 136 functions with P=5.96×10^−10^), the quantity of cells (rank 11, P=1.02×10^−9^), the activation of leukocytes (rank 15, P=3.68×10^−9^) and cytotoxicity (rank 19, P=6.25×l0^−9^). Downstream Effects Analysis (as defined in IPA) for visualization of the biological trends in this study revealed that the proliferation of immune cells and quantity of cells were predicted to decrease in the third- compared to the first-trimester pNK cells (Fig. 5). By contrast, the activation of leukocytes and cytotoxicity were predicted to increase in the third- compared to the first-trimester pNK cells (Fig. 5). The combined network demonstrated that many genes in the data set were upregulated (e.g., *1L1R1* and *CEACAM1*) and that KIR genes were downregulated (Fig. 5).

### In silico analysis of miRNA-mRNA interactions in pNK cells during pregnancy

**Figure 6:**
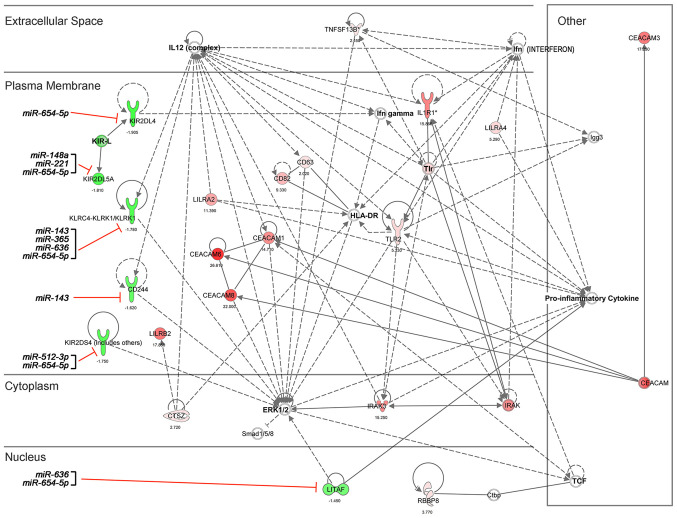
Ingenuity Pathway Analysis (IPA) Core Analysis-based network of microRNA (miRNA)-mRNA interactions in peripheral blood NK (pNK) cells during pregnancy. The network displays interactions between natural killer (NK) cell function-associated genes that were differentially expressed between first- and third-trimester pNK cells; the network was filtered by NK cell context with a minimum of ten genes per category. Genes upregulated in third- relative to first-trimester pNK cells are indicated in shades of red; genes downregulated in third-trimester pNK cells are indicated in shades of green; genes in white circles were not in our data set but were inserted by IPA because they are connected to this network. Numbers below symbols represent the fold changes in transcript level of third- relative to first-trimester pNK cells. Solid and dashed lines indicate direct and indirect interactions, respectively. miRNAs that showed an upregulated expression in third- compared to first-trimester pNK cells and had the *in silico* target sites in the 3′-untranslated region (3′-UTR) of downregulated genes (green) are also shown.

As upregulated miRNAs promoted mRNA degradation or repression in pNK cells during pregnancy, a subset of downregulated genes would include miRNA target candidates. To identify these targets as well as effector miRNAs, we used IPA and the microRNA.org database (http://www.microrna.org/microrna/getMirnaForm.do) to search for over-represented miRNA seed sequences. We identified 27 seeds originating from the 12 miRNAs that were significantly upregulated in the third- compared to the first-trimester pNK cells (Table I). At least one miRNA target site was identified in 69% (9 of 13) of the downregulated NK cell function-related genes, with an average of three miRNA target sites per gene. Note that the target site of one upregulated C19MC miRNA, *miR-512-3p*, was present in the 3′-UTR of the upregulated *KIR2DS4* gene. The mRNA-miRNA network that was filtered by NK cell context with a minimum of 10 genes per category is depicted in Fig. 6. One miRNA, *miR-7-2#*, which was significantly downregulated in the third- compared to the first-trimester cells did not have the target sites of upregulated NK cell function-associated genes during pregnancy.

## Discussion

The miRNAs within the human imprinted C19MC, a primate-specific miRNA cluster encompassing 46 miRNA genes in the human genome, are expressed exclusively in the placenta; i.e., placenta-associated miRNAs (16–18). Placenta-associated miRNAs are expressed in human villous trophoblasts and are secreted into the maternal circulation via exosomes (19). During pregnancy, exosomes are involved in cell-to-cell communication between the placenta and peripheral blood immune cells. Taylor *et al* reported that placenta-derived exosomes are present in the maternal circulation and suppress T cells with exosome-associated proteins (e.g., Fas ligand) (20). Although it is likely that exosomal C19MC miRNAs contribute to placental-maternal communication, few studies have examined whether exosomal placenta-associated miRNAs modulate the expression of their targets in recipient maternal immune cells. Recently, we demonstrated the exosome-mediated transfer of a placenta-associated miRNA, *miR-517a-3p*, into maternal pNK cells with the subsequent modulation of a target, *PRKG1* (13). We hypothesized that placenta-associated miRNAs, as well as proteins (i.e., KLRK1 ligands) may be transferred via exosomes from placental trophoblasts into maternal NK cells and modulate gene expression, which possibly contributes to the maintenance of pregnancy.

In the present study, we performed comprehensive miRNA and gene expression profiling of maternal pNK cells using a combination of real-time PCR-based array and DNA micro-array analyses. In particular, we focused on placenta-associated C19MC miRNAs in pNK cells and found that C19MC miRNAs were incorporated into maternal pNK cells during pregnancy (Figs. 1^3^). Moreover, the levels of incorporated C19MC miRNAs were increased in the pNK cells in a gestational stage-dependent manner and decreased rapidly following delivery (Figs. 1^3^). These results are consistent with those of our previous study, showing that the levels of incorporated *miR-517a-3p* and *miR-518b* differed significantly before and after delivery (13). These results, together with data from our previous study, provide evidence that the transfer of placental C19MC miRNAs into maternal pNK cells occurs during pregnancy.

With respect to the transcriptional level, we observed differential gene expression between the first- and third-trimester pNK cells on DNA microarray analysis. The differentially expressed genes included many NK cell function-associated genes (Table I). IPA Core Analysis revealed that many cell cycle regulation pathways were included in high-ranking canonical pathways with the upregulation of cell cycle- and proliferation-related genes (Fig. 4). For example, the estrogen-mediated S-phase entry pathway was significantly altered as the highest-ranking canonical pathway. Estrogens induce the proliferation of estrogen receptor-positive cells by stimulating G1/S transition and the subsequent progression of the cell cycle (21). NK cells express estrogen receptors (22). The number of uterine NK cells increase by the elevation of plasma estrogen levels during pregnancy (23). In the present study, cell cycle regulation pathways showed a trend toward cell cycle progression in pNK cells during pregnancy. These pathways may be of interest for further study.

On the other hand, the interpretation of other functions in pNK cells affected by pregnancy may be more complex than that of the cell cycle. The network generated by IPA Downstream Effects Analysis revaled complex prediction of the effects of gene expression changes in the third- compared to the first-trimester pNK cells (Fig. 5). For example, the downregulation of *KIR2DL4* and *KIR3DL1* indicates a prediction of leading to the activation of NK cell cytotoxicity. Yusa *et al* examined the inhibitory functions of the KIR2DL4 cytoplasmic domain using NK-like cell lines that had a chimeric receptor of the KIR2DL4 cytoplasmic domain fused to the extracellular and transmembrane domains of this chimeric receptor KIR3DL1 and revealed that both receptors exhibited similar capacity to inhibit cytotoxicity (24). Both KIR2DL4 and KIR3DL1 molecules can decrease the cytotoxicity of NK cells. In the network, the upregulation of *CEACAM1*, a member of the carcinoembryonic antigen gene family, indicates a prediction of leading to the activation of leukocytes. Lu *et al* investigated Ceacam1 as a regulator of graft-vs.-host disease and graft-vs.-tumor after allogeneic bone marrow transplantation in mouse models, and demonstrated that Ceacam1 regulated T-cell activation and the number of donor T cells in lymphoid organs and graft-vs.-host disease target tissues (25). In the network, the upregulation of *LILRB2* indicates a prediction of leading to the inhibition of immune cell proliferation. Human LILRB2 inhibits NK T-cell activation by directly binding to CD1D and preventing antigen loading, thus leading to reduction of T-cell proliferation (26–28). In the network, another upregulated LILR gene, *LILRB3*, indicates a prediction of leading to the inhibition of cell quantity. Older *Lilrb3*^−/−^ mice have been shown to have an increased number of peritoneal B1 B lymphocytes (29), indicating that the upregulation of Lilrb3 decreased the number of B1 B cells. Taken together, it is inferred from IPA Downstream Effects Analysis that gene expression changes in pNK cells may contribute to the increase in the cytotoxicity of third-compared to first-trimester pNK cells. In addition to NK cell cytotoxicity, pNK cells may also influence adaptive immune responses by acting on dendritic cells and T/B cells, and NK cells may be involved in controlling the proliferation of these immune cells. However, it should be noted that the changes in expression of genes indicated with yellow lines in the network shown in Fig. 5 were inconsistent with the predicted expression changes of these genes with respect to NK functions generated in this study.

NK cells consist of two distinct subsets based on the levels of expression of the low-affinity Fc γ receptor IIIA (CD16) and neural cell adhesion molecule (CD56) receptors. CD56^dim^ CD16^pos^ NK cells predominantly mediate cytotoxic effector functions (i.e., natural cytotoxic activity and antibody-dependent cellular cytotoxicity), while CD56^bright^ CD16^neg^ NK cells primarily produce cytokines. The pNK cells are mainly CD56^dim^ CD16^pos^ (30,31). The majority of pNK cells appear to produce low levels of cytokines, but show a high-level expression of KIRs. Thus, pNK cells are potent cytotoxic effector cells. In this study, an activating KIR gene *(KIR2DS4)* and activating receptor NKG2-D type II integral membrane protein gene *(KLRK1)* showed a significantly decreased expression during pregnancy, and inhibitory KIR genes (*KIR2DL4, KIR2DL5A*, and *KIR3DL1*) simultaneously showed a decreased expression (Table I). By contrast, the expression levels of both an activating LILR gene (*LILRA2*) and inhibitory LILR genes (*LILRB2* and *LILRB3*) in the third-trimester pNK cells were significantly higher than those in the first-trimester cells (Table I). These differentially expressed genes of immune receptors did not represent a universal shift toward the same direction of functional response of pNK cells to cytotoxicity. However, previous studies have suggested that NK cells can adapt their responsiveness to a changing environment *in vivo* (32,33). It is possible that pNK cells adjust to different gestational conditions during pregnancy.

In conclusion, the present study provides details of the miRNA and gene expression profiles in pNK cells during pregnancy. PCR-based array analysis indicated that 25 miRNAs, including six placenta-derived miRNAs (C19MC miRNAs), were significantly upregulated in the third- compared to the first-trimester pNK cells. pNK cells incorporated C19MC miRNAs, and these interactions were possibly mediated via exosomes. The rapid clearance of C19MC miRNAs also occurred in the NK cells following delivery. DNA microarray analysis revealed that 69 NK cell function-related genes were differentially expressed between the first- and third-trimester pNK cells. Pathway and network analysis suggested that these gene expression changes of pNK cells contribute to the increase in the cytotoxicity, as well as the cell cycle progression of third-trimester pNK cells. Thirteen of the 69 NK cell function-related genes were significantly downregulated between the first- and third-trimester pNK cells. Nine of the 13 downregulated NK function-associated genes were *in silico* target candidates of 12 upregulated miRNAs, including C19MC miRNA *miR-512-3p*. Further *in vitro* experiments are required to validate the NK cell function-related genes as real targets of miRNAs identified in this study. Although recent studies have revealed that miRNAs regulate fundamental NK cell processes, including cytotoxicity, cytokine production and proliferation (34,35), the functions of placental C19MC miRNAs in maternal plasma also remain to be elucidated. Such studies are currently in progress in our laboratory. Further characterization of the subsets of maternal NK cells and the discovery of target genes of C19MC miRNAs may provide further insight into the understanding of maternal NK cell functions (i.e., the establishment and maintenance of pregnancy, and parturition outbreak mechanism) during pregnancy.

## Figures and Tables

**Table I:** NK cell function-related genes that were differentially expressed between the first- and third-trimester pNK cells in DNA microarray analysis.

NK cell function	Gene symbol	Log fold change (3rd vs. 1st)	P-value	miRNA^a^
Cytolytic pathway	*CTSZ*	1.44	0.0025	
Cytolytic pathway	*TNFSF13B*	1.29	0.0009	
Cytolytic pathway	*TNFAIP2*	3.26	0.0009	
Cytokines, chemokines and their receptors	*CXCR7 (CMKOR1)*	−1.06	0.0009	
Cytokines, chemokines and their receptors	*IL13RA1*	3.22	0.0053	
Cytokines, chemokines and their receptors	*IRF2BP2*	0.76	0.0130	
Cytokines, chemokines and their receptors	*IL17RA*	1.01	0.0067	
Secretory signature, cell cycle, proliferation	*CDKN3*	2.74	0.0009	
Secretory signature	*HM13*	0.83	0.0013	
Secretory signature	*DEGS1*	1.07	0.0012	
Secretory signature	*GLRX*	0.72	0.0017	
Cell surface and adhesion molecules	*ITM2C*	2.57	0.0009	
Cell surface and adhesion molecules	*CD59*	2.18	0.0009	
Cell surface and adhesion molecules	*CD244 (2B4A, 2B4)*	−0.70	0.0018	*miR-143*
Cell surface and adhesion molecules	*CD24*	4.59	0.0010	
Cell surface and adhesion molecules	*CD82*	3.22	0.0009	
Cell surface and adhesion molecules	*CD63*	1.02	0.0016	
Cell surface and adhesion molecules	*CD302*	3.57	0.0009	
Cell surface and adhesion molecules	*CD200R1*	1.81	0.0082	
Cell surface and adhesion molecules	*KIR2DL5A*	−0.86	0.0000	*miR-148a, miR-221, miR-654-5p*
Cell surface and adhesion molecules	*KIR3DL1*	−0.84	0.0009	*miR-221, miR-654-5p*
Cell surface and adhesion molecules	*KIR2DS4*	−0.80	0.0003	*miR-512-3p, miR-654-5p*
Cell surface and adhesion molecules	*KLRK1 (NKG2D, CD314)*	−0.83	0.0051	*miR-143, miR-365, miR-636, miR-654-5p*
Cell surface and adhesion molecules	*KIR2DL4*	−0.93	0.0009	*miR-654-5p*
Cell surface and adhesion molecules	*LILRA2*	3.51	0.0009	
Cell surface and adhesion molecules	*LILRA4*	2.40	0.0050	
Cell surface and adhesion molecules	*LILRA5*	2.65	0.0009	
Cell surface and adhesion molecules	*LILRA6*	3.58	0.0014	
Cell surface and adhesion molecules	*LILRB2*	4.16	0.0009	
Cell surface and adhesion molecules	*LILRB3*	2.58	0.0015	
Cell surface and adhesion molecules	*CEACAM1 (BGP, CD66A)*	3.87	0.0015	
Cell surface and adhesion molecules	*CEACAM6 (CD66C)*	4.73	0.0009	
Cell cycle, proliferation	*TFDP1*	1.19	0.0009	
Cell cycle, proliferation	*MCM6*	1.12	0.0012	
Cell cycle, proliferation	*E2F1*	1.78	0.0009	
Cell cycle, proliferation	*GTSE1*	2.31	0.0010	
Cell cycle, proliferation	*CKS2*	1.04	0.0009	
Cell cycle, proliferation	*MKI67*	3.45	0.0011	
Cell cycle, proliferation	*PCNA*	1.13	0.0016	
Cell cycle, proliferation	*CHEK1*	1.86	0.0009	
Cell cycle, proliferation	*CKS1B*	1.11	0.0008	
Cell cycle, proliferation	*CDK2*	0.73	0.0095	
Cell cycle, proliferation	*KNTC1*	0.96	0.0009	
Cell cycle, proliferation	*MCM2*	1.41	0.0009	
Cell cycle, proliferation	*CCNB1*	1.60	0.0009	
Cell cycle, proliferation	*CCNE2*	2.15	0.0009	
Cell cycle, proliferation	*E2F2*	3.35	0.0009	
Cell cycle, proliferation	*CCNB2*	3.61	0.0009	
Cell cycle, proliferation	*KPNA2*	0.65	0.0009	
Cell cycle, proliferation	*RBBP8*	1.91	0.0009	
Cell cycle, proliferation	*CCNA2*	2.83	0.0009	
Cell cycle, proliferation	*GADD45A*	1.99	0.0011	
Cell cycle, quiescence	*FBXO9*	1.28	0.0011	
Cell cycle, quiescence	*DHRS7*	−0.25	0.0044	
JAK/STAT pathway	*JAK2*	0.61	0.0009	
JAK/STAT pathway	*SLA2*	−0.99	0.0022	
JAK/STAT pathway,	*IL1R1*	3.99	0.0024	
NF-κB pathway regulation				
JAK/STAT pathway	*IL6R*	3.43	0.0017	
TGF-β pathway	*RUNX2*	1.08	0.0052	
TGF-β pathway	*BMP2*	2.49	0.0019	
TGF-β pathway	*BMPR1A*	−1.22	0.0068	*miR-143, miR-654-5p, miR-922*
P25K pathway	*AKT2*	−0.55	0.0082	*miR-143, miR-148a, miR-193a-5p, miR-221, miR-345, miR-365, miR-574-3p, miR-654-5p, miR-922*
P43K pathway	*FOXM1*	2.83	0.0009	
P50K pathway	*PAK1*	1.95	0.0009	
NF-κB pathway regulation	*IRAK3*	3.93	0.0010	
NF-κB pathway regulation	*TLR2*	2.41	0.0024	
NF-κB target genes	*DUSP2*	−1.05	0.0058	
NF-κB target genes	*LITAF*	−0.54	0.0049	*miR-636, miR-654-5p*
NF-κB target genes	*MARCKS*	4.25	0.0009	

a miRNAs that showed an upregulated expression in the third- compared to the first-trimester pNK cells and had the *in silico* target sites in the 3′-UTR of downregulated genes. NK, natural killer; pNK, peripheral blood NK; miRNA, microRNA; 3′-UTR, 3′-untranslated region.

**Table II:** Gene-related functions with a minimum of ten genes per category in the IPA defined Diseases and Bio Functions category as revealed by IPA Core Analysis.

Category	Function	Disease or function annotation	P-value	P-value ranking of 136 gene-related functions	P-value ranking filtered by NK cell context	Gene^a^
Cellular development, cellular growth and proliferation, hematological system development and function	Proliferation	Proliferation of immune cells	5.96×l0^−10^	8	1	***CCNA2, CD24, CD244, CD59, CDK2, CEACAM1, CTSZ, E2F1, E2F2, GADD45A, IL13RA1,IL1R1, IL6R, JAK2***, **KIR2DS4** (includes others), KLRC4-***KLRK1/KLRK1, LILRB2, LILRB3, TLR2, TNFSF13B***
Tissue morphology	Quantity	Quantity of cells	1.02×l0^−9^	11	2	ACKR3, ***AKT2, BMP2, BMPR1A, CCNA2, CCNB1, CCNB2, CD200R1, CD244, CD59, CD63, CDK2, E2F1, E2F2, FOXM1, GADD45A, IL13RA1, IL17RA, IL1R1, IL6R, IRAK3, JAK2, LILRB3, LITAF, RBBP8, RUNX2, TFDP1, TLR2, TNFSF13B***
Cell-to-cell signaling and interaction, hematological system development and function, immune cell trafficking, inflammatory response	Activation	Activation of leukocytes	3.68×l0^−9^	15	3	***CD244, CD59, CD63, CEACAM1***, CEACAM3, ***CEACAM6, GADD45A, IL1R1, IL6R, JAK2, KIR3DL1***, KLRC4-***KLRK1/KLRK1, LILRA2, LILRB2, LILRB3, SLA2, TLR2, TNFSF13B***
Cell death and survival	Cytotoxicity	Cytotoxicity	6.25×l0^−9^	19	4	***CD244, CD59, CEACAM1, CHEK1, IL1R1, KIR2DL4,KIR2DS4*** (includes others),***KIR3DL1* KLRC4-*KLRK1/KLRK1,PAK1, TLR2***
Cell death and survival, cellular compromise	Toxicity	Toxicity of cells	4.13×10^−8^	28	5	***CD244, CD59, CEACAM1, IL1R1, KIR2DL4, KIR2DS4*** (includes others), ***KIR3DL1***, KLRC4-***KLRK1/KLRK1, PAK1, TLR2***
Cell-to-cell signaling and interaction, hematological system development and function, immune cell trafficking, inflammatory response	Activation	Activation of mononuclear leukocytes	4.47×l0^−8^	29	6	***CD244, CD59, CEACAM1, CEACAM6, GADD45A, IL6R, KIR3DL1***, KLRC4-***KLRK1/KLRK1, LILRA2, LILRB2, L1LRB3, SLA2, TLR2, TNFSF13B***
Cell-to-cell signaling and interaction, hematological system development and function, immune cell trafficking, inflammatory response	Activation	Activation of lymphocytes	1.82×l0^−7^	38	7	***CD244, CD59, CEACAM1, CEACAM6, GADD45A, IL6R, KIR3DL1***, KLRC4-***KLR*K1*/KLRK1, LILRB2, LILRB3, SLA2, TLR2, TNFSF13B***
Cell-to-cell signaling and interaction, hematological system development and function, immune cell trafficking, inflammatory response	Activation	Activation of T lymphocytes	5.90×10^−7^	44	8	***CD244, CD59, CEACAM1, CEACAM6, GADD45A, IL6R, KIR3DL1***, KLRC4-***KLRK1IKLRK1, LILRB2, SLA2, TLR2***
Cellular function and maintenance	Homeostasis	Lymphocyte homeostasis	1.34×10^−6^	53	9	***AKT2, CCNB2, CD244, CDK2, CHEK1, E2F1, E2F2, IL17RA, IL1R1, LILRB3, RUNX2, TLR2, TNFSF13B***
Cell-mediated immune response, cellular function and maintenance, hematological system development and function	Homeostasis	T cell homeostasis	4.69×10^−6^	63	10	***AKT2, CCNB2, CD244, CDK2, CHEK1, E2F1, E2F2, IL17RA, IL1R1, RUNX2, TLR2, TNFSF13B***
Hematological system development and function, tissue morphology	Quantity	Quantity of lymphocytes	6.38×10^−6^	67	11	ACKR3, ***CCNB2, CD244, E2F1, E2F2, GADD45A, IL13RA1, IL17RA, IL1R1, JAK2, LILRB3, RUNX2, TLR2***, TNFSF13B
Immunological disease	Systemic autoimmune syndrome	Systemic autoimmune syndrome	1.20×10^−5^	77	12	***CCNB1, CD24, CD244, CDK2, CEACAM1***, CEACAM8, ***DUSP2, E2F1, IL17RA, IL1R1, IL6R, IRAK3, JAK2, LILRB3, TLR2, TNFSF13B***
Connective tissue disorders, inflammatory disease, skeletal and muscular disorders	Rheumatic disease	Rheumatic disease	1.59×10^−5^	80	13	***BMP2, CCNB1, CD200R1, CD24, CD244, CD59, CDK2, CEACAM1***, CEACAM8, ***DUSP2, IL17RA, IL1R1, IL6R, JAK2, KIR3DL1, TLR2, TNFSF13B***
Cell-to-cell signaling and interaction	Communication	Communication of cells	1.11×10^−4^	108	14	***AKT2, BMP2, CD244, CD59, CEACAM1, CEACAM6, IL13RA1, IL1R1, JAK2, KIR2DL4***, KLRC4-***KLRK1/KLRK1, LILRA2, LILRB2, LILRB3, LITAF, PAR1, SLA2, TLR2, TNFSF13B***
Connective tissue disorders, inflammatory disease, skeletal and muscular disorders	Arthritis	Arthritis	3.42×10^−4^	118	15	***BMP2, CCNB1, CD200R1, CD24, CD244, CD59, DUSP2, IL17RA, IL1R1, IL6R, JAK2, KIR3DL1, TLR2, TNFSF13B***
Cancer	Breast or colorectal cancer	Breast or colorectal cancer	2.44×10^−3^	136	16	ACKR3, ***AKT2***, ***BMP2***, ***BMPR1A***, ***CCNA2***, ***CCNB1***, ***CCNB2***, ***CD24***, ***CD244***, ***CDK2***, ***CDKN3***, ***CEACAM1***, ***CEACAM6***, CEACAM8, ***CHEK1***, ***CTSZ***, ***DHRS7***, ***DUSP2***, ***FOXM1***, ***GLRX***, ***HM13***, ***IL17RA***, ***IL6R***, ***IRF2BP2***, ***JAK2***, ***KIR2DL4***, ***KIR3DL1***, ***KNTC1***, ***KPNA2***, ***LILRA6***, ***LILRB3***, ***MCM6***, ***MKI67***, ***PAK1***, ***PCNA***, ***RBBP8***, ***RUNX2***, ***TFDP1***, ***TLR2***, ***TNFAIP2***

a Genes written in bold font were NK cell function-associated genes; genes not written in bold font were not in our data set but were inserted by IPA as they are connected to this network. IPA, Ingenuity Pathway Analysis; NK, natural killer.
